# Early-Stage High-Concentration Thiacloprid Exposure Induced Persistent Behavioral Alterations in Zebrafish

**DOI:** 10.3390/ijerph191710920

**Published:** 2022-09-01

**Authors:** Zhongtang Xie, Guanghua Lu, Yeting Yu

**Affiliations:** Key Laboratory of Integrated Regulation and Resources Development of Shallow Lakes of Ministry of Education, College of Environment, Hohai University, Nanjing 210098, China

**Keywords:** thiacloprid, hypoactivity, neurotoxicity, neurotransmitter, depressive-like

## Abstract

As a major neonicotinoid insecticide, thiacloprid (THCP) is frequently detected in aquatic environments worldwide due to its heavy use, posing potential threats to aquatic organisms. In this study, zebrafish (*Danio rerio*) embryos were exposed to THCP (1, 10, 100, 1000 and 10,000 μg/L) for 5 days and then recovered in THCP-free water for 20 days to investigate the effects of early-stage THCP exposure on the development, antioxidant defense, and neurotransmitter systems of zebrafish, and explore their recovery mechanism. The results show that THCP exposure induced developmental toxicity and oxidative stress in zebrafish. The hypoactivity, behavioral alterations (decreased avoidance and edge preference behaviors) and neurotoxicity were found throughout the exposure-recovery experiments. THCP exposure altered the expression of γ-aminobutyric acid (GABA)- and serotonin (5-HT)-related genes accompanied by the decrease in GABA and 5-HT contents. However, after recovery, GABA content returned to the control level, but 5-HT did not, indicating that only the serotonergic system was persistently disrupted. Overall, our results suggest that the disruption of the serotonergic system and oxidative stress may aggravate neurotoxicity and that the former was the main reason for the depressive-like behavior. This study could help to unravel the mechanisms of the behavioral alterations induced by early-stage THCP exposure in zebrafish.

## 1. Introduction

In the past two decades, neonicotinoids have become the most widely used insecticide groups worldwide, mainly targeting numerous piercing-sucking and some certain chewing pests [1]. Neonicotinoids exert a high degree of selectivity for nicotinic acetylcholine receptors (nAChRs) in the central nervous system of insects [2]. Compared to target invertebrates, neonicotinoids bind with relatively less affinity to nAChRs in vertebrates [3]. This property has led to neonicotinoids becoming favored and popular insecticides worldwide over the past three decades [1]. Nevertheless, in recent years, neonicotinoids have been reported to have adverse effects on non-target organisms such as crustaceans, birds, fishes, and mammals [4,5,6,7]. For most neonicotinoids, due to their high solubility, they enter the freshwater environment easily in many ways, such as through rainfall, sewage and snowmelt [8,9]. Moreover, neonicotinoids are persistent enough to reach coastal areas and eventually enter the marine environment [10]. It was found that contamination with neonicotinoids in coastal areas was mainly from adjacent riverine discharges, which could pose a relatively high threat to freshwater and marine organisms [11]. Thus, the potential effects of neonicotinoids on aquatic ecosystems have drawn increasing attention.

When referring to neonicotinoids, imidacloprid is definitely the first one that people think of, since it was the first neonicotinoid to enter the market and was most widely used worldwide [3]. However, with the increase in restrictions on the use of imidacloprid, the usage of other neonicotinoids, such thiacloprid (THCP), increased due to its low toxicity to bees compared to nitroguanidine neonicotinoids [12]. As a major neonicotinoid insecticide, THCP was introduced into the market in 2001 and subsequently detected in aquatic environments worldwide. In Yarramundi Lagoon near Sydney, the THCP concentration was up to 1.37 μg/L [13]. In surface waters of southern Ontario, Canada, the THCP concentration reached 427 ng/L [14]. To this day, the highest concentration of THCP was detected in the Elbe River near Hamburg, reaching 4.5 μg/L [15]. It was reported that field-realistic water concentrations of THCP could reduce the abundance and biomass of aquatic insects [16]. In addition, THCP was also proven to affect ontogeny and growth rate, induce hepatotoxicity and alter the expression of apoptotic and immune-related genes in fish [17,18,19].

The nAChRs of vertebrates are part of the cys-loop family of neurotransmitter-gated ion channels, including γ-aminobutyric acid (GABA), serotonin (5-HT) and glycine receptors [3]. GABA is the main inhibitory neurotransmitter in the central nervous system of vertebrates, produced by GABAergic neurons and released through synapses, and then endogenously activating GABA receptors to modulate neurotransmission [20,21]. 5-HT is the principal neurotransmitter for modulating various brain functions and psychiatric conditions such as depression and antisocial behavior [22,23]. The alteration of genes that are involved in GABA and 5-HT synthesis, metabolism and receptor trafficking may result in dysfunction of the corresponding neurotransmitter systems [22,24,25]. It was reported that two typical neonicotinoids, imidacloprid and thiamethoxam, could induce abnormality in locomotor activity, alter the expression of genes that are involved in GABA and 5-HT transmission, and cause neurotoxicity in zebrafish [26]. However, besides the locomotor activity, imidacloprid and thiamethoxam may induce some other behavioral effects on non-target organisms when they have neurotransmitter system abnormalities, although these were not examined in previous studies. In addition, whether THCP induces such effects like imidacloprid and thiamethoxam remains unknown.

Zebrafish (*Danio rerio*) is widely used as a model vertebrate for toxicological researches, with rapid development and high transparency that enables researchers to study the toxicity of chemicals at the early stage of zebrafish [27]. The developmental nervous system, whatever the species, is vulnerable to exogenous chemicals. This is because some key developmental processes were tightly orchestrated at the early stage of organisms, such as proliferation, migration and synaptogenesis and so on, so that any disruption of these processes may lead to the failure of normal development of nervous system [28]. The aims of the present study were to investigate the growth and development toxicity, neurotoxicity, antioxidant response, behavioral changes and neurotransmitter system abnormalities of zebrafish induced by early-life exposure to THCP, and to explore the recovery mechanism of abnormal behavior (avoidance response and edge preference) and motor activity changes through exposure recovery experiments.

## 2. Materials and Methods

### 2.1. Chemicals

THCP (purity > 99.4%) was provided by Shanghai Pesticide Research Institute Co., Ltd. (Shanghai, China). Dimethyl sulfoxide (DMSO; purity > 99.9%) and phosphate buffered saline (PBS) were obtained from Amresco (Solon, OH, USA).

### 2.2. Exposure-Recovery Experiments of Zebrafish Embryos

Zebrafish embryos (AB strain, 2 h post-fertilization, hpf) and embryo culture media were sourced from Nanjing EzeRinka Biotechnology Co., Ltd. (Nanjing, China). The experiment consisted of two phases: a THCP-exposure phase (5 days) and a recovery phase (20 days). The exposure solution was obtained by diluting the THCP stock solution dissolved in DMSO with embryo culture media. The final DMSO concentration was 0.01% (*v*/*v*). A solvent control (SC) was composed of culture media and 0.01% (*v*/*v*) DMSO. A blank control (BC) was composed of culture media. Embryos were randomly divided into seven groups (350 embryos per group), including one BC group, one SC group, and five THCP treatment groups (1, 10, 100, 1000 and 10,000 μg/L, respectively), and raised at 28 ± 1 °C on a 14 h light/10 h dark cycle for 5 days. In our previous study, we exposed adult zebrafish to different concentrations of THCP (at 1, 4.5, 10 and 45 μg/L) for 4 days, and we found that these ‘low’ concentrations of THCP did not induce death and oxidative stress in the zebrafish, but 100 μg/L and higher concentrations of THCP induced hepatotoxicity [19]. Although these low concentrations of THCP did not induce biochemical responses in adult zebrafish, we speculated that low concentrations of THCP (1 and 10 μg/L) may induce some detrimental effects on the larval zebrafish because they are more sensitive when facing chemicals compared to the adult zebrafish. Each group was replicated three times simultaneously. The exposure solution was completely replaced once a day. When replacing water, zebrafish embryo or larvae were carefully collected with a pipette and immediately transferred into new six-well plates containing fresh exposure solutions. The actual concentration of THCP was determined during the exposure period (Table S1 in the Supplementary Materials). At the end of exposure, 100 larvae were collected from each group and anesthetized in ice-cold water. Then, the collected samples were immediately frozen in liquid nitrogen and stored at −80 °C for qRT-PCR and biochemical analysis. The remaining larvae were transferred into THCP-free dechlorinated carbon-filtered tap water for 20 days of recovery, and raised under the same light conditions as embryos and fed with live paramecia twice daily. Half of the water was replaced daily. After 5 days of recovery, 30 larvae were collected. After 20 days of recovery (by the end of the recovery), the rest of the larvae were collected. All experimental procedures received approval from the Animal Ethics Committee of “Hohai University”.

### 2.3. Quantification of THCP in Exposure Solution

The actual concentrations of THCP in exposure solution were determined after each solution renewal (0 h) and before next solution renewal (24 h). One milliliter of water samples was collected from each treatment group and filtered by a 0.22 μm Millipore membrane filter. THCP concentration was measured by ultra-performance liquid chromatography connected to a Xevo triple quadrupole mass spectrometer (UPLC-MS/MS) (Waters, Milford, MA, USA). The UPLC separation was performed using an ACQUITY UPLC BEH C18 analytical column (2.1 mm × 100 mm, 1.7 μm). The mass spectrometer was operated in the multiple reaction-monitoring mode. The mobile phase consisted of water/methanol with 0.1% formic acid (mobile phase A, *v*/*v*, 98:2) and acetonitrile (mobile phase B) run at a flow rate of 0.4 mL/min. The injection volume was 5 μL. The limits of detection and quantification for THCP were 8.80 ng/L and 29.4 ng/L, respectively, and the recoveries of THCP were 87.4–101%. 

### 2.4. Growth and Developmental Toxicity

During the exposure period, the heart rate, hatching rate and body length of larvae were recorded at 48 hpf, 72 hpf and 120 hpf, respectively. The survival rate and malformation rate were measured at 120 hpf. The representative malformation of zebrafish is shown in Figure S1 in the Supplementary Materials. There were no significant differences between the BC group and SC group in all parameters, so we only used the SC group as the control in the following experiments.

### 2.5. Locomotor Activity Measurement

The locomotor activity of zebrafish larvae was monitored by a video-track system (ViewPoint Life Sciences Inc., Lyon, France) without any noise interference at days 0, 5 and 20 during the recovery period. Each treatment group was tested with 24 replicates. One larva was randomly chosen from each group, transferred into 24-well plates and adapted for 10 min under the laboratory condition. Then, the locomotor activity of larvae was recorded under light conditions for 15 min. The average swimming speed (ASS), maximum acceleration (MMA) and percentage of low-mobility time (PLT) of the larvae under light condition were calculated.

### 2.6. Five-Fish Behavioral Assay

The five-fish behavioral assay was performed as previously described [29,30]. The visual stimuli were the key element of this assay. They were created in Microsoft PowerPoint (PPT, Microsoft, Redmond, WA, USA) according to a previous PPT template [29]. In our PPT, a moving red disc was set in the upper half of the well, which moved from left to right and back over a straight trajectory, and a stationary red disc was set in the bottom half of the well (see details in the Supplementary Materials). The stationary disc can counter-balance the brightness and red color of the moving disc. Red (RGB, 255, 0, 0) was chosen because it can be easily removed in the following image assay. According to a previous study, it is the moving subject that stimulates larvae rather than color [29]. The PowerShot G7 X Mark II camera was used to image larvae in this assay. Briefly, five zebrafish larvae, kept in their respective treatment groups, were placed in each well of six-well plates. The internal sampling method was used to record the locations of larvae—specifically, images were collected every 30 s for 30 min. During the first 15 min, larvae were imaged on a light background without visual stimuli. During the second 15 min, the same larvae were imaged with visual stimuli. It was reported that a moving disc was an aversive stimulus for larvae, and larvae would swim away from it and show an edge preference (thigmotaxis) [29]. Specific analyses of avoidance behavior and thigmotaxis were conducted in the following image analysis.

### 2.7. Image Analysis in ImageJ

ImageJ (http://rsb.info.nih.gov/ij/ (accessed on 1 June 2022)) was used to analyze the pictures obtained in the five-fish behavioral assay. The ImageJ macro used in this analysis was modified from a previously published macro [30]. Specific steps are shown in Figure S2 in the Supplementary Materials. After running the macro, ImageJ generated the results containing well numbers, data regarding the *x* and *y* coordinates of larvae’s centroids, and the coordinates of the well’s midpoint. Microsoft Excel was used to process these data. If the y coordinates of larvae were larger than those of a well’s midpoint, the larvae were considered to be in the bottom half of the well (‘down’). If not, the larvae were considered to be in the upper half of the well (‘up’). If the distance from the larva to the well’s midpoint was larger than the square root of the radius of the well, the larva was considered to be on the edge. If not, the larva was considered to be in the center area of the well. The effects of THCP on avoidance behavior and thigmotaxis of larvae were investigated by determining how often the larvae swam ‘down’ away from visual stimuli and how often the larvae were located on the edge of the well, respectively. 

### 2.8. Biochemical Analysis

The activities of superoxide dismutase (SOD), catalase (CAT), glutathione S-transferase (GST) and acetylcholinesterase (AChE) and the contents of malondialdehyde (MDA) and total protein were measured by using commercial assay kits from Jiancheng Bioengineering Institute (Nanjing, China). Protein concentration was measured using the Braford assay [31]. SOD, CAT and GST activities and MDA content in zebrafish larvae exposed to THCP for 5 days were measured. The samples were added to normal saline according to a mass (g):volume (mL) ratio of 1:9. Then, the samples were homogenized (3500× *g*, 1 min) and centrifuged (4000× *g*, 10 min) at 4 °C. The supernatant was collected for the biochemical assay. SOD activity was determined at 550 nm by determining its inhibitory ability according to the reduction of nitro blue tetrazolium [32]. CAT activity was determined at 405 nm as previously described [33]. GST activity was determined at 412 nm by measuring its catalytic ability of binding reduced glutathione (GSH) to 1-chloro-2, 4-dinitorbenzene [34]. MDA content was determined using the thiobarbituric acid method at 532 nm [35]. 

The larvae on days 0, 5 and 20 of recovery were used to determine GABA and 5-HT contents and AChE activity. GABA and 5-HT were measured at 450 nm by using diagnostic enzyme-linked immunosorbent assay kits purchased from Jiangsu Meimian industrial Co., Ltd. (Yancheng, China), as described in the Supplementary Materials. The AChE activity was measured at 412 nm by following the increase in yellow color that was generated from thiocholine when it reacted with 5, 5′-dithiobis-2-nitrobenzoate [36]. All assays were performed according to the manufacturer’s instructions. 

### 2.9. Gene Expression Measurement

GABA-related genes (*gad1b*, *abat*, *gat1*, *gabra1* and *glsa*), 5-HT related genes (*tph2*, *mao*, *slc6a4a* and *htr1aa*) and *ache* (encodes AChE) were analyzed using qRT-PCR. The total RNA of 50 larvae was extracted using TRIzol reagent (Life Technologies, Gaithersburg, MD, USA). RNA concentrations and the 260/280 and 260/230 ratios were measured using a NanoDrop 2000 (Thermo Fisher Scientific, Waltham, MA, USA). RNA purity was assessed using gel electrophoresis. cDNA was obtained using the PrimeScript™ RT reagent Kit with gDNA Eraser (Takara, Dalian, China). Then, qRT-PCR was conducted on an ABI PCR9700 (Applied Biosystems, Foster City, CA, USA). Relative gene expressions were calculated using the 2^−^^ΔΔCt^ method [37]. Primer sequences of target genes are shown in Table S2. 

### 2.10. Statistical Analysis

Statistical analysis was performed using SPSS 23.0 (SPSS Inc, Chicago, IL, USA). All the data are expressed as mean ± standard deviation (SD). Normality of data and homogeneity of variance were tested using the Shapiro–Wilk test and Levene’s test, respectively. When the data were from different treatment groups, one-way analysis of variance (ANOVA) followed by Tukey’s post hoc tests was used. When the data were analyzed between two groups, the independent-sample t-test was used. *p* < 0.05 was considered statistically significant. GraphPad Prism 8 (GraphPad Software Inc., San Diego, CA, USA) was used for constructing graphs.

## 3. Results

No THCP was detected in the control groups. The actual concentrations of THCP during the exposure periods deviated between 15.10% and 18.94% from the nominal concentrations, showing good agreement. Thus, subsequent analyses were based on the nominal concentrations. 

### 3.1. Growth and Developmental Toxicity

**Figure 1:**
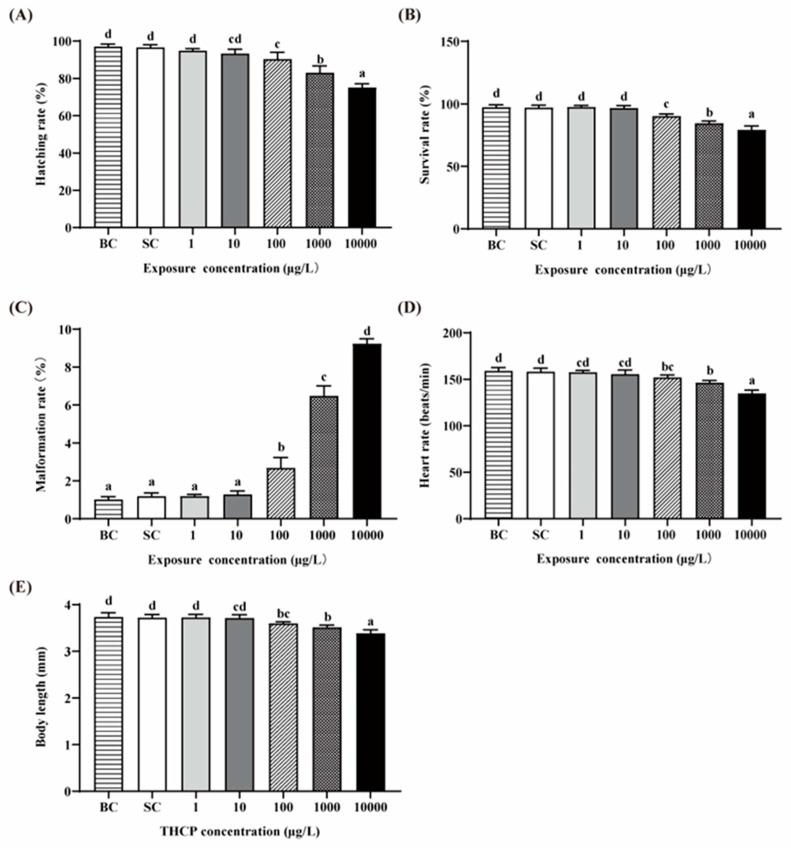
Effects of THCP on zebrafish larvae in different stages. (**A**) Hatching rate, (**B**) survival rate, (**C**) malformation rate, (**D**) heart rate and (**E**) body length of zebrafish. One-way ANOVA followed by Tukey’s post hoc tests was used to determine statistical differences. Different letters indicate significant differences between different treatment groups (*p* < 0.05).

There were no significant differences in all indexes between the BC and SC groups (Figure 1A–E). Thus, we used the SC as the control in the following analyses. The hatching, survival, malformation and heart rates and the body length of zebrafish exposed to two lower concentrations of THCP (1 and 10 μg/L) were not different from those in the SC groups, while significant decreases in the hatching, survival and heart rates and body length were observed in the higher concentration groups (100, 1000 and 10,000 μg/L), showing concentration-dependent manners. The malformation rate was significantly increased by 1.24-, 4.44- and 6.75-fold by 100, 1000 and 10,000 μg/L THCP, respectively. Overall, a higher concentration had adverse effects on the development of larvae. 

### 3.2. Effects of THCP on Larval Locomotor Activity

**Figure 2:**
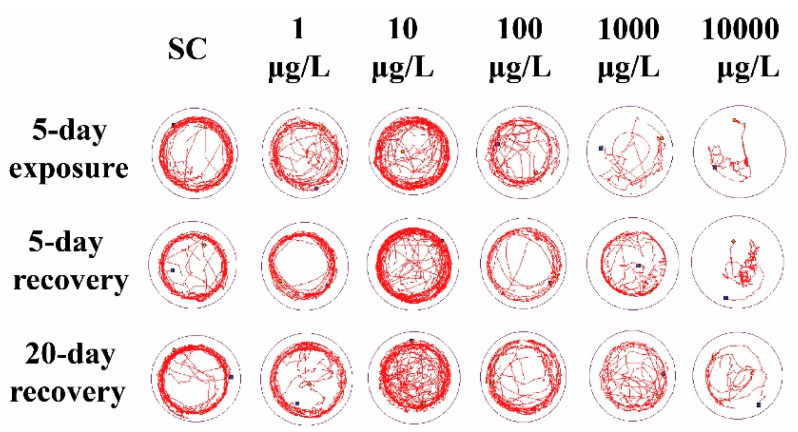
The representative locomotor trajectory in 5 min.

Compared to the SC group, 1 μg/L THCP exposure did not cause significant alteration in the locomotor trajectory (Figure 2), while 10 μg/L THCP exposure led to more chaotic trajectory. With the increase in THCP concentration from 100 to 10,000 μg/L, the locomotor trajectory of larvae became much sparser compared to the SC group and showed obvious concentration dependence. After THCP exposure, zebrafish larvae showed a preference for swimming in the center of the well, with fewer trails on the edge of the well. Even after 5 days or 20 days of recovery, this preference remained. 

**Figure 3:**
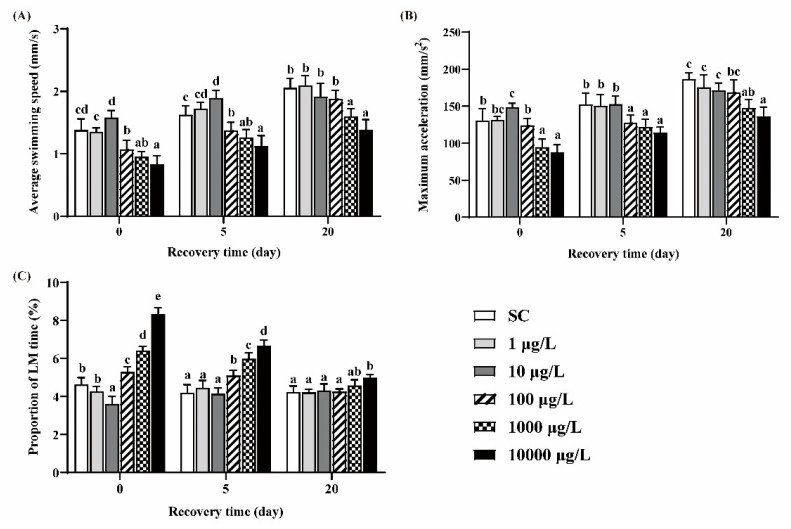
The ASS (**A**), MMA (**B**) and PLT (**C**) of zebrafish larvae under light conditions. One-way ANOVA followed by Tukey’s post hoc tests was used to determine statistical differences. Different letters indicate statistically significant differences between different treatment groups at the same time (*p* < 0.05).

Zebrafish larvae in the 10 μg/L group exhibited significant higher ASS and MMA and lower PLT, while larvae in the 100, 1000 and 10,000 μg/L groups exhibited significant lower ASS and MMA and higher PLT, compared to the SC group (Figure 3A–C). After 5 days of recovery, these behavioral parameters still did not return to the levels of those in the SC group. After 20 days of recovery, ASS, MMA and PLT in the 100 μg/L group returned to the control levels, while those in the two highest concentration groups could not recover.

### 3.3. Larval Avoidance Behavior and Edge Preference

**Figure 4:**
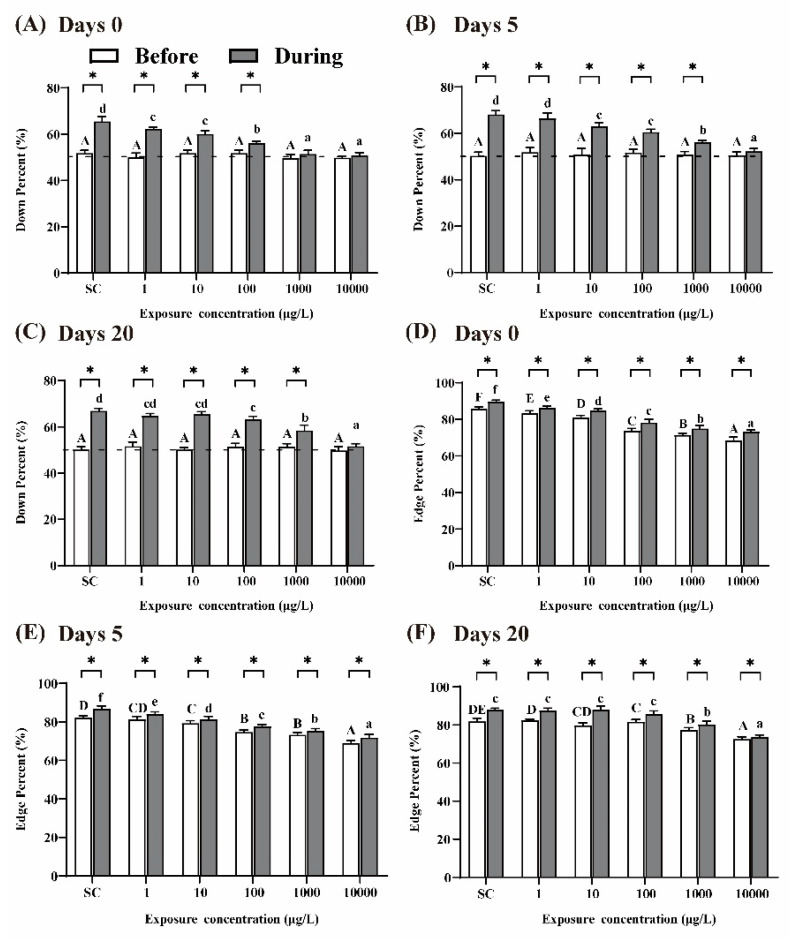
Avoidance behavior (**A**–**C**) and edge preference (**D**–**F**) of larvae at days 0, 5 and 20 of recovery. ‘Before’ and ‘During’ represent prior to the visual stimuli and in the presence of the visual stimuli, respectively. One-way ANOVA followed by Tukey’s post hoc tests were used to determine statistical differences. Capital letters and lowercase letters were labelled to distinguish ‘Before the visual stimuli’ and ‘During the visual stimuli’. Different letters indicate significant differences between different treatment groups (*p* < 0.05). In the same group, asterisk indicates statistically significant difference between ‘Before the visual stimuli’ and ‘Before the visual stimuli’. (*p* < 0.05). Independent-sample *t*-test was used to compare two independent groups.

It was reported that larvae display preferences for the edge of the well and swim ‘down’ in the presence of visual stimuli [30]. After 5 days of THCP exposure (i.e., at day 0 of recovery), prior to the visual stimuli, the percentages of larvae swimming ‘down’ in the well were approximately 50% in all the groups, which were agreed with the expectation from random 50:50 distribution (Figure 4A). In the presence of visual stimuli, the percentages of larvae swimming ‘down’ in the well significantly increased in the SC group, indicating that normal larvae were sensitive to the visual stimuli. Compared to the SC group, with the increase in THCP concentration, the percentages of larvae swimming ‘down’ in the THCP treatment groups significantly decreased in a concentration-dependent manner, which indicated that THCP induced inhibitory effects on the avoidance behavior of larvae. After 5 or 20 days of recovery (Figure 4B,C), the inhibitory effects induced by THCP were still observed in the 100, 1000 and 10,000 μg/L groups, indicating that high-THCP concentration exposure induced long-term effects on the avoidance response of the larvae.

The larvae in the SC group exhibited a strong preference for the edge of the well during the whole experimental period (Figure 4D–F). In the presence of visual stimuli, the ‘edge’ percentage was significantly increased in all the groups compared to that before the visual stimuli, indicating that visual stimuli could promote larvae’s edge preference. With the increase in THCP concentration, edge preference in response to the visual stimuli was significantly decreased in the THCP-treated groups in a concentration-dependent manner, indicating that THCP suppressed this preference. After 5 and 20 days of recovery, edge preference of larvae in the 1000 and 10,000 μg/L groups did not return to the control level, suggesting that a high concentration of THCP induced persistent effects on edge preference. 

### 3.4. Changes of GABA, 5-HT Contents and AChE Activity

**Figure 5:**
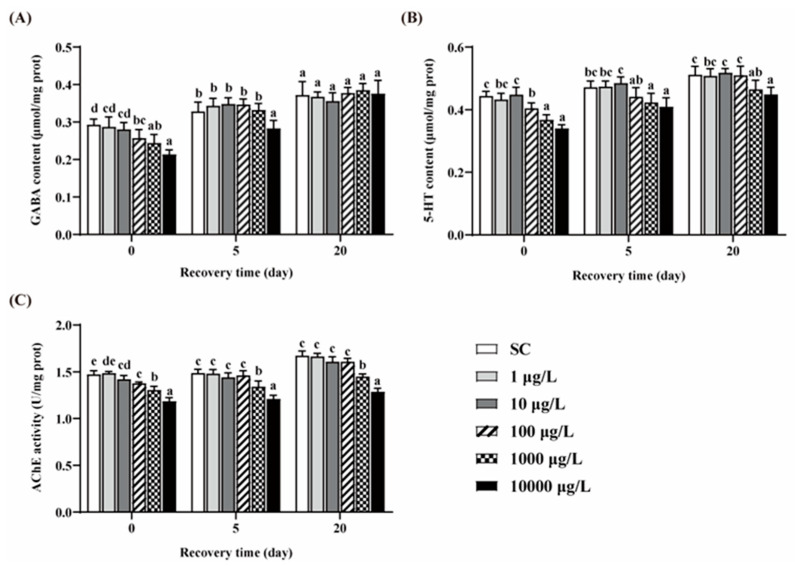
GABA (**A**) and 5-HT (**B**) contents, and AChE (**C**) activity of zebrafish. One-way ANOVA followed by Tukey’s post hoc tests were used to determine statistical differences. Different letters indicate significant differences between different treatment groups at the same time (*p* < 0.05).

Compared to the SC group, no significant differences in the contents of GABA and 5-HT were observed in the two lower concentrations of THCP groups (Figure 5A,B). After 5 days of THCP exposure, the GABA and 5-HT contents in the three higher concentrations groups were significantly lower than those in the SC group. After 5 days of recovery, GABA content in the 100 and 1000 μg/L group returned to the control level, but failed in the 10,000 μg/L group. The content of 5-HT returned to the control level in the 100 μg/L group, but failed in the higher concentration groups. After 20 days of recovery, there were no significant differences in GABA content between THCP exposure and the SC group. However, the 5-HT content in the 1000 and 10,000 groups was still lower than that in the SC group. These results indicate that the effects of high concentrations of THCP on zebrafish during early life may be reversible for GABAergic system, while irreversible for serotoninergic system.

THCP exposure significantly decreased AChE activity at all concentrations (Figure 5C). After 5 and 20 days of recovery, AChE inhibition caused by the two highest concentration of THCP could not be restored. 

### 3.5. Neurotransmitters-Related Genes Expressions

From the above sections, we found that two lower concentrations of THCP (1 and 10 μg/L) did affect the phenotypes such as growth and development, behaviors, and neurotransmitter contents. However, as for behavioral alterations, a high concentration of THCP decreased ASS and MMA and increased PLT during the exposure and recovery periods, indicating that THCP induced irreversible hypoactivity in zebrafish. Thus, we chose 100, 1000 and 10,000 μg/L of THCP for gene expression analyses.

**Figure 6:**
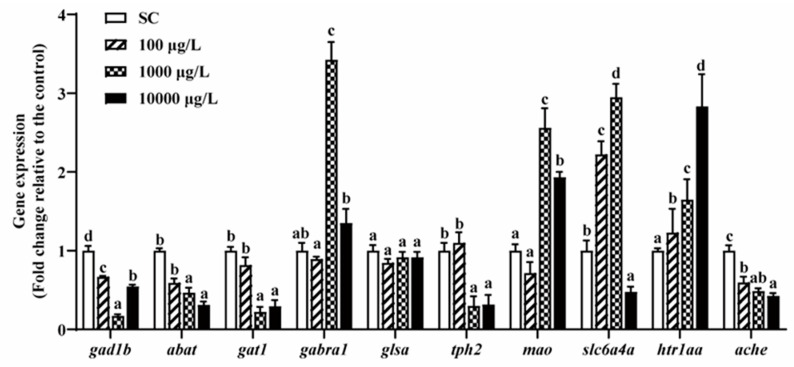
The expression of GABA-, 5-HT-, and AChE-related genes in zebrafish larvae after THCP exposure. One-way ANOVA followed by Tukey’s post hoc tests were used to determine statistical differences. Different letters indicate significant differences between different treatment groups (*p* < 0.05).

After 5 days of THCP exposure, the expression of genes related to GABA, 5-HT and AChE was significantly altered with one exception of *glsa* (Figure 6). In the GABAergic system, 1000 and 10,000 μg/L THCP significantly down-regulated the expression of *gat1* and up-regulated the expression of *gabra1*, while *abat* and *gad1b* expression was significantly down-regulated by all of the THCP treatments. In the serotonergic system, 1000 and 10,000 μg/L of THCP significantly down-regulated *tph2* expression and up-regulated *mao* expression. The expression of *slc6a4a* was significantly up-regulated at 100 and 1000 μg/L but down-regulated at 10,000 μg/L. Furthermore, THCP exposure significantly up-regulated *htr1aa* expression and down-regulated AChE expression in a concentration-dependent manner. 

### 3.6. THCP Induced Oxidative Stress in Zebrafish Larvae

**Figure 7:**
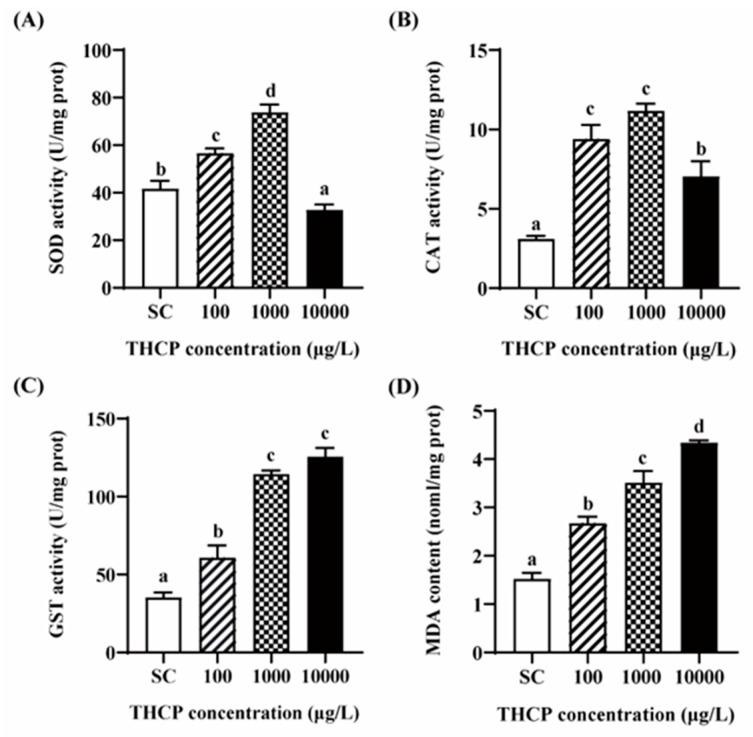
THCP-induced oxidative stress in larvae. (**A**) SOD activity. (**B**) CAT activity. (**C**) GST activity. (**D**) MDA content. One-way ANOVA followed by Tukey’s post hoc tests were used to determine statistical differences. Different letters indicate statistically significant differences between different treatment groups (*p* < 0.05).

From Figure 7, SOD and CAT activities were significantly increased in the 100 and 1000 μg/L groups compared to the SC group. In the highest concentration group, SOD activity was significantly inhibited compared to the control, while the induction level of CAT was lower than that in the two lower concentrations. GST activity was increased by 1.72-, 3.24- and 3.56-fold in the 100, 1000 and 10,000 μg/L groups, respectively. In addition, compared to the SC group, MDA contents were significantly increased by THCP, with 1.75-, 2.30- and 2.85-fold induction corresponding to 100, 1000 and 10,000 μg/L, respectively. In general, all indicators of oxidative stress showed obvious concentration–response relationships.

## 4. Discussion

The hatching, survival, malformation and heart rates and body length at the early stage of zebrafish are often used to assess the growth and developmental toxicity of chemicals [38,39]. In this study, from the indexes mentioned above, we confirmed that high concentrations of THCP (over 100 μg/L) induced growth and developmental toxicity in zebrafish. Similar results were observed in some previous neonicotinoid-related studies. For instance, THCP exposure led to lower growth and delay in the development of the common carp (*Cyprinus carpio*) [18]. Environmentally relevant concentrations of dinotefuran (a neonicotinoid) were reported to adversely affect the growth of zebrafish [40]. In addition, acetamiprid (a neonicotinoid) exposure could decrease the hatching, survival and heart rates, and increase malformation rate of zebrafish, which were consistent with our results [41].

The alterations of locomotor activity at the early stage of zebrafish can be used to evaluate the developmental neurotoxicity induced by chemicals [42]. Thus, the hypoactivity of larvae observed in our study could be a sign of neurotoxicity, which was also found in imidacloprid and thiamethoxam-exposed zebrafish [26]. The hypoactivity of fish may lead to the reduction in feeding activity, body growth and the ability to evade predation, ultimately affecting their survival [43].

As we know, zebrafish larvae will avoid large moving objects, and this behavior results from a predator avoidance mechanism [44,45]. The normal function of the predator avoidance mechanism was disrupted after THCP exposure, as evidenced by the reduced avoidance behavior and thigmotaxis. Depressive-like behavior of zebrafish is generally reflected by reduced motor activity and exploratory behavior and increased freezing behavior [21]. In addition, it was reported that decreases in AChE activity could increase depression-like behaviors [46]. In this study, hypoactivity and reduced avoidance behavior and thigmotaxis, accompanied by AChE inhibition, suggested the occurrence of depressive-like behavior in larvae exposed to high concentrations of THCP.

Generally, the dysfunction of neurotransmitter systems is the basis of neurobehavioral disorders in zebrafish larvae, because the precursors of the neurotransmitters are sensitive to toxicants [47,48,49]. GABA-mediated neurotransmission starts at the early stage of fish development, which begins after the patterning of the spinal cord and hindbrain, regulating the circuitry underlying locomotor behaviors (e.g., rhythmic swimming response) [50]. *Gad1b* encodes glutamic acid decarboxylase, converting glutamate into GABA [51]. *Abat* encodes the key rate-limiting enzyme for GABA degradation [52]. GAT1 (encoded by *gat1*), a GABA transporter, is expressed on the plasma membrane of neurons and astrocytes and is responsible for 85% of GABA reuptake [53]. Glutaminase (encoded by *glsa*) converts glutamine into glutamate [54]. In this study, the down-regulation of *gad1b, abat* and *gat1*, accompanied by the decreased GABA content, indicated that GABAergic system was disrupted after THCP exposure. No significant alteration of *glsa* expression was observed, indicating that THCP may not affect the synthesis of the raw material of GABA. GABA A receptor (encoded by *gabra1*) is universally found in mature neurons, taking part in mediating majority of fast inhibitory transmission [51]. It was reported that the up-regulation of *gabra1* can suppress stress- or anxiety-related behavior of zebrafish [55]. The up-regulation of *gabra1* may reflect the self-adjustment of zebrafish when the depression-like behavior occurred.

5-HT plays a crucial role at the early stage of zebrafish neurodevelopment, which is considered as a neurotrophic factor during embryogenesis and a modulator in neurotransmitter system [56]. Disturbances in serotonin signaling at the early stage of vertebrates may lead to neurodevelopmental and neuropsychiatric disorders [57]. *Htr1aa* (encodes 5-HT receptor) is broadly expressed in both larval and adult zebrafish [58]. *Slc6a4a* encodes the transporter of 5-HT [58]. In this study, the up-regulation of *htr1aa* and down-regulation of *slc6a4a* induced by highest concentration of THCP indicated that 5-HT neurotransmission was disrupted by high THCP exposure at the early stage of zebrafish. *Tph2* encodes tryptophan hydroxylase-2 for 5-HT synthesis [59]. Monoamine oxidase (encoded by *mao*) was responsible for the degradation of 5-HT [56]. The up-regulation of *mao* and down-regulation of *tph2* in this study indicated that the synthesis and degradation of 5-HT might be affected, which was supported by the decrease in 5-HT content. A lack of endogenous 5-HT during central nervous system development affects the proper wiring of brain, which may produce persistent alterations causing neurodevelopmental disorders [60]. In addition, the depletion of 5-HT has been shown to result in less movement of zebrafish, which was consistent with our result [61,62]. Overall, our results indicate that the serotonergic system may participate in the regulation of the locomotor activity and depressive-like behavior after THCP exposure. The dysfunction of serotonergic neurons at the development stage has been demonstrated to be associated with psychiatric diseases such as depression and schizophrenia [63]. By the end of recovery period, depressive-like behavior was still observed in zebrafish exposed to a high concentration of THCP during their early life, suggesting that this abnormal behavior may be associated with the persistent disruption of the serotonergic system. This disruption affected neurotransmission and neurodevelopment, thus leading to neurotoxicity, at least partially. We confirmed that developmental THCP exposure induced neurobehavioral defects in zebrafish that remained effective in adolescence, which was in line with a previous study showing that developmental imidacloprid exposure caused long-lasting neurobehavioral impairments in juvenile and adult zebrafish [64].

Oxidative stress leads to an imbalance between reactive oxygen species (ROS) generation and the ability of the antioxidant system in organisms, which occurs when the antioxidant system could not eliminate excessive ROS [65]. MDA, the final product of lipid peroxidation, can be used as an indicator of oxidative stress. Antioxidant enzymes, such as CAT, SOD and GST, contribute to the elimination of ROS and play significant roles in the detoxification process in fish. In this study, with the increase in THCP concentration, GST increased in a concentration-dependent manner, while SOD and CAT activities were first increased in the 100 and 1000 μg/L groups and then decreased in the 10,000 μg/L group. In the highest concentration group, SOD activity was significantly lower than that in the SC group, while CAT activity was significantly higher than that in the SC group. Similar alterations to SOD and CAT activities were observed after dinotefuran or thiamethoxam exposure, which were consistent with our results [40,66]. Previous studies showed that mild oxidative stress can induce the increase in GST activity, which is beneficial to cope with oxidative damage [67,68]. Generally, the activities of antioxidant enzymes increase when the antioxidant system is activated to eliminate ROS. However, when ROS generation increases to a breaking point where the antioxidant system is overwhelmed, the activities of antioxidant enzymes decrease [69]. The alterations of antioxidant enzymes and the increase in MDA observed in this study indicated that the fish underwent oxidative stress. A previous study reported that ROS production was a major contributor to the developmental neurotoxicity, and the detrimental effects on the neurobehavior could be induced by several factors such as altered gene expression in the central nervous system, impairments in motor neurons and increases in oxidative damage [70].

AChE is often used as a biomarker of neurotoxicity induced by toxicants. At the early stage of zebrafish development, AChE is required for the development of neurons and muscle [71]. The down-regulation of *ache* was matched by the decrease in AChE activity in the present study, resulting in the accumulation of ACh in the brain, which then affected the normal function of the nervous system. The vulnerability of the central nervous system to oxidative stress may lead to developmental brain defects, and subsequently induce developmental neurotoxicity and affect the swimming ability of zebrafish larvae [72]. A previous study showed that oxidative stress, neurotoxicity and the reduction in body length were responsible for the hypoactivity induced by microplastics in zebrafish [73]. Similarly, in this study, the hypoactivity of zebrafish may be attributed to growth and developmental toxicity, oxidative stress and neurotoxicity induced by the early-stage THCP exposure.

## 5. Conclusions

Early-stage THCP exposure inhibited the growth and development of zebrafish induced oxidative stress, and led to persistent hypoactivity of larvae. In addition, the decreased locomotor activity, avoidance behavior and edge preference were observed even after 20 days of recovery, suggesting that the larvae have persistent depressive-like behavior, which can be attributed to the disruption of the serotonergic system rather than the GABAergic system based on alterations to the corresponding neurotransmitter contents during the recovery period. Moreover, we found that alterations to the serotonergic system and oxidative stress may aggravate neurotoxicity. Considering that the locomotor activity and behavioral responses are critical for food acquisition and predator avoidance, impairment of these behaviors can, therefore, reduce the ability of fish to survive in aquatic ecosystems.

## Data Availability

The data showed in this study are available on request from the corresponding author.

## Supplementary Materials

The following supporting information can be downloaded at: https://www.mdpi.com/article/10.3390/ijerph191710920/s1, Principle of the assay. Figure S1: The malformations observed in the THCP-exposed zebrafish larvae at 96 hpf.; Figure S2: ImageJ macro was used to automatically analyze the behavior of larvae; Table S1: Actual concentrations of THCP in exposure solutions; Table S2: Primers of target genes that were analyzed in the study.
