# Supplementary material for: Early-Stage High-Concentration Thiacloprid Exposure Induced Persistent Behavioral Alterations in Zebrafish
Source: Int J Environ Res Public Health. 2022 Sep 1;19(17):10920. doi: 10.3390/ijerph191710920 (PMC9518391; doi:10.3390/ijerph191710920)
Supplement: Supplementary file 1 [file ijerph-19-10920-s001.zip › ijerph-1873904-supplementary/Supplementary Materials/Supplementary Material.pdf]

Early-stage high-concentration thiacloprid exposure induced persistent behavioral alterations in zebrafish

Zhongtang Xie, Guanghua Lu*, Yeting Yu

Key Laboratory of Integrated Regulation and Resources Development of Shallow Lakes of Ministry of Education, College of Environment, Hohai University, Nanjing 210098, China

*Corresponding author: Guanghua Lu

E-mail address: ghlu@hhu.edu.cn

Tel.: +86-025-8378-6406

Principle of the assay

1. Fish γ-aminobutyric acid (GABA) ELISA kit

This kit is used to determine the content of fish GABA by double antibody sandwich method. Purified fish GABA capture antibody was coated with microtiter plate wells to prepare solid-phase antibody. Fish GABA capture antibody was successively added into the microporous coated plate, and then combined with HRP-labeled detection antibody to form antibody-antigen-enzyme-labeled antibody complex. After washing completely, substrate 3,3′,5,5′-Tetramethylbenzidine (TMB) is added and incubated for color development. TMB is converted to blue under the catalysis of HRP enzyme, and to the final yellow under the action of acid. The depth of color was positively correlated with fish GABA content in the sample. Color change in reaction was measured by absorbance at 450 nm with a microplate reader. GABA content was calculated by standard curve.

2. Fish serotonin (5-HT) ELISA kit

This kit is used to determine the content of fish 5-HT by double antibody sandwich method. Purified fish 5-HT capture antibody was coated with microtiter plate wells to prepare solid-phase antibody. Fish 5-HT capture antibody was successively added into the microporous coated plate, and then combined with HRP-labeled detection antibody to form antibody-antigen-enzyme-labeled antibody complex. After washing completely, substrate 3,3′,5,5′-Tetramethylbenzidine (TMB) is added and incubated for color development. TMB is converted to blue under the catalysis of HRP enzyme, and to the final yellow under the action of acid. The depth of color was positively correlated with

fish 5-HT content in the sample. Color change in reaction was measured by absorbance at 450 nm with a microplate reader. 5-HT content was calculated by standard curve.

The malformations of zebrafish

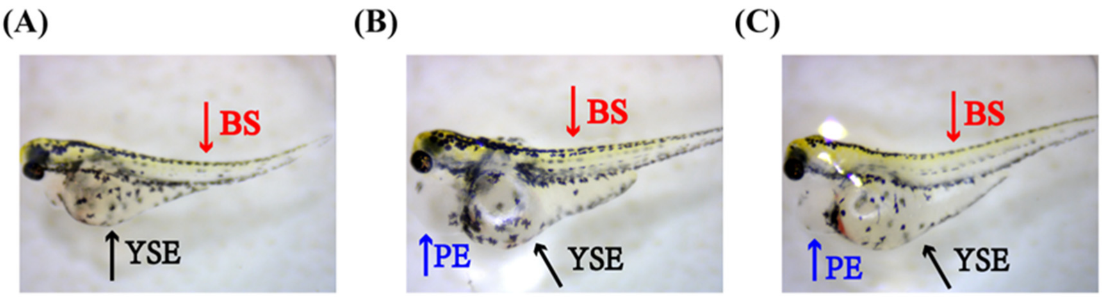

Figure S1. The malformations observed in the THCP-exposed zebrafish larvae at 96 hpf. (A–C) Three representative larvae were selected to show the malformations. BS, bent spine; YSE, yolk sac edema; PE, pericardial edema.

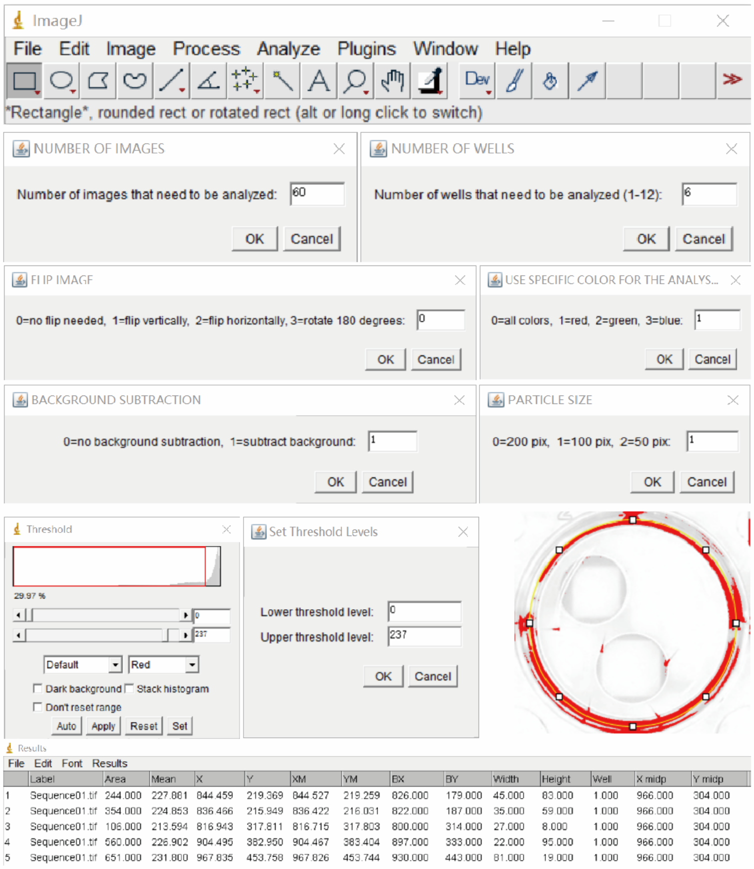

Figure S2. ImageJ macro was used to automatically analyze the behavior of larvae.

Table S1. Actual concentrations of THCP in exposure solutions.

Exposure concentrations	1 μg/L	10 μg/L	100 μg/L	1000 μg/L	10000 μg/L
0 h（μg/L）	0.94 ±0.07	9.92±0.08	99.62±0.17	997.28±4.28	9982.37±16.23
24 h（μg/L）	0.82±0.09	8.37±0.13	81.34±0.28	840.61±8.39	8192.11±23.94

Data are expressed as the mean ± SD of three replicates. Exposure solutions were collected at 0 h (after each solution renewal) and 24 h (before each solution renewal).

Table S2. Primers of target genes that analyzed in the study.

Gene	Sequence of the primer (5' - 3') Forward	Sequence of the primer (5' - 3') Reverse	PE (%)
gat1 (GABA transporter)	TGTCAGCTGGCAAAAACTTG	ACAAAGGTCCCAGTGGAGTG	102.2
abat (4-aminobutyrate aminotransferase)	GTGACGCTAATGCAAAAATGAAG	CGATGTCCTGCAGAGTGGTG	94.2
gabra1 (GABA receptor)	GCAGTGGCAATGACCAGATA	GGAAAACTCCGACTGTCTGC	95.3
gad1b (glutamate decarboxylase 1b)	TGGCGTCCCAGGTAGATAAT	ACCGTACATCTCCACGAAGG	90.6
glsa (glutaminase)	CAGCAAGGTTGACAACGAGA	CGAAGGTTGTGTTGGGAGTT	88.9
tph2 (tryptophan hydroxylase 2)	AAGACCTGATTCTGCGACTG	TAGGCTGAGACACCTTTACG	93.1
slc6a4a (solute carrier family 6 member 4a)	TGAAATACAGAGCAAGGCAACC	ACAGGAGGGCGATGAACAC	93.3
mao (monoamine oxidase)	AGTTCTTCTGACCACACGCAAT	CCTGTTCTGGGAGTTTGTGC	93.8
htr1aa (5-hydroxytryptamine receptor 1 A a)	AGACCGCTGCCCATCACTA	AGAGGAAATCTGCCTCCACT	94.0
ache (acetylcholinesterase)	CCCAGACATCAGGGAGTGAT	CACCGATCCAGACGGAGTAT	90.8
GAPDH	CTGGTGACCCGTGCTGCTT	TTTGCCGCCTTCTGCCTTA	100.9

PE refers to PCR efficiencies.
