# Supplementary figures and images for: Early-Stage High-Concentration Thiacloprid Exposure Induced Persistent Behavioral Alterations in Zebrafish
Source: Int J Environ Res Public Health. 2022 Sep 1;19(17):10920. doi: 10.3390/ijerph191710920 (PMC9518391; doi:10.3390/ijerph191710920)

## Slide 1
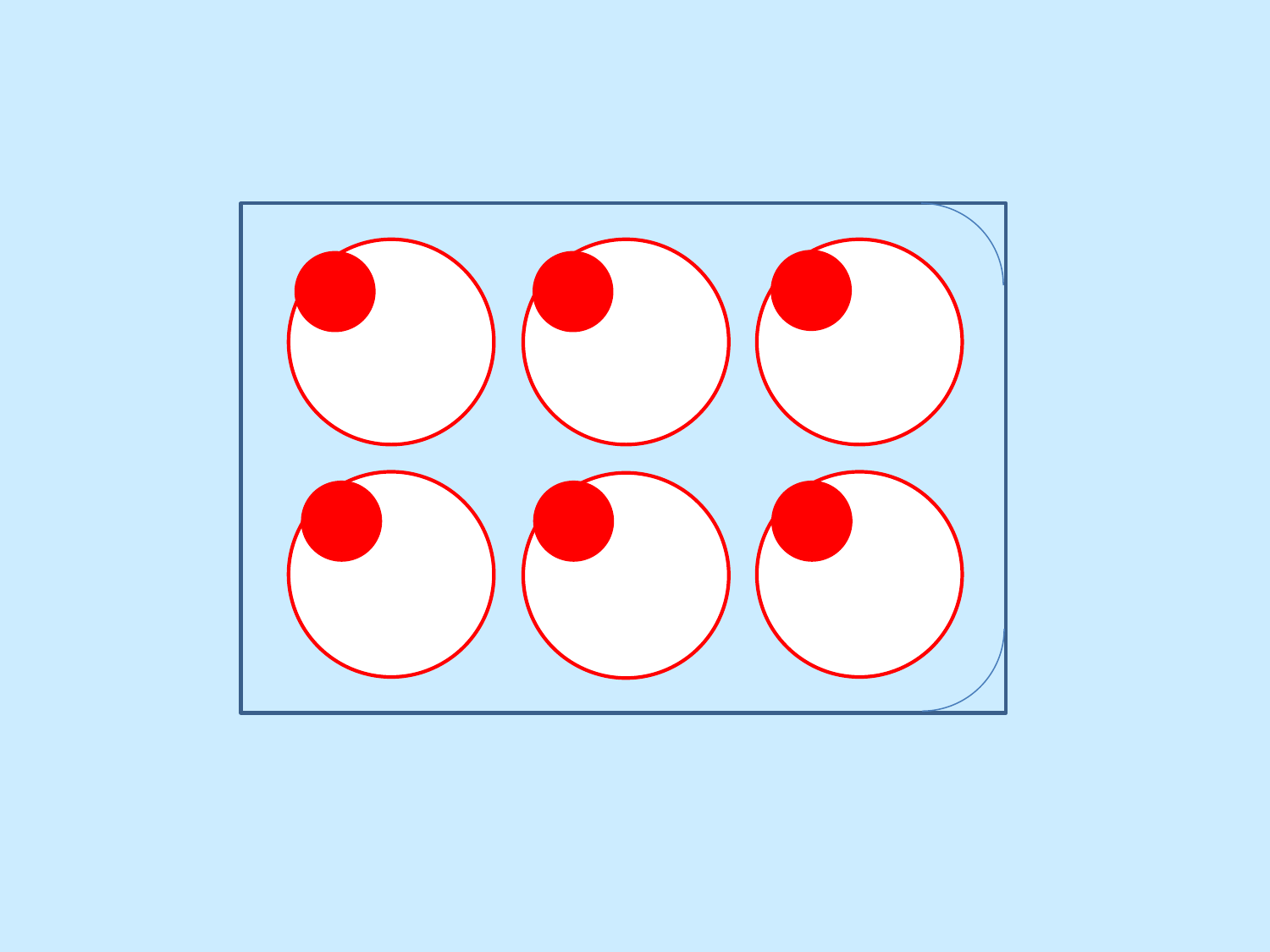

## Slide 2
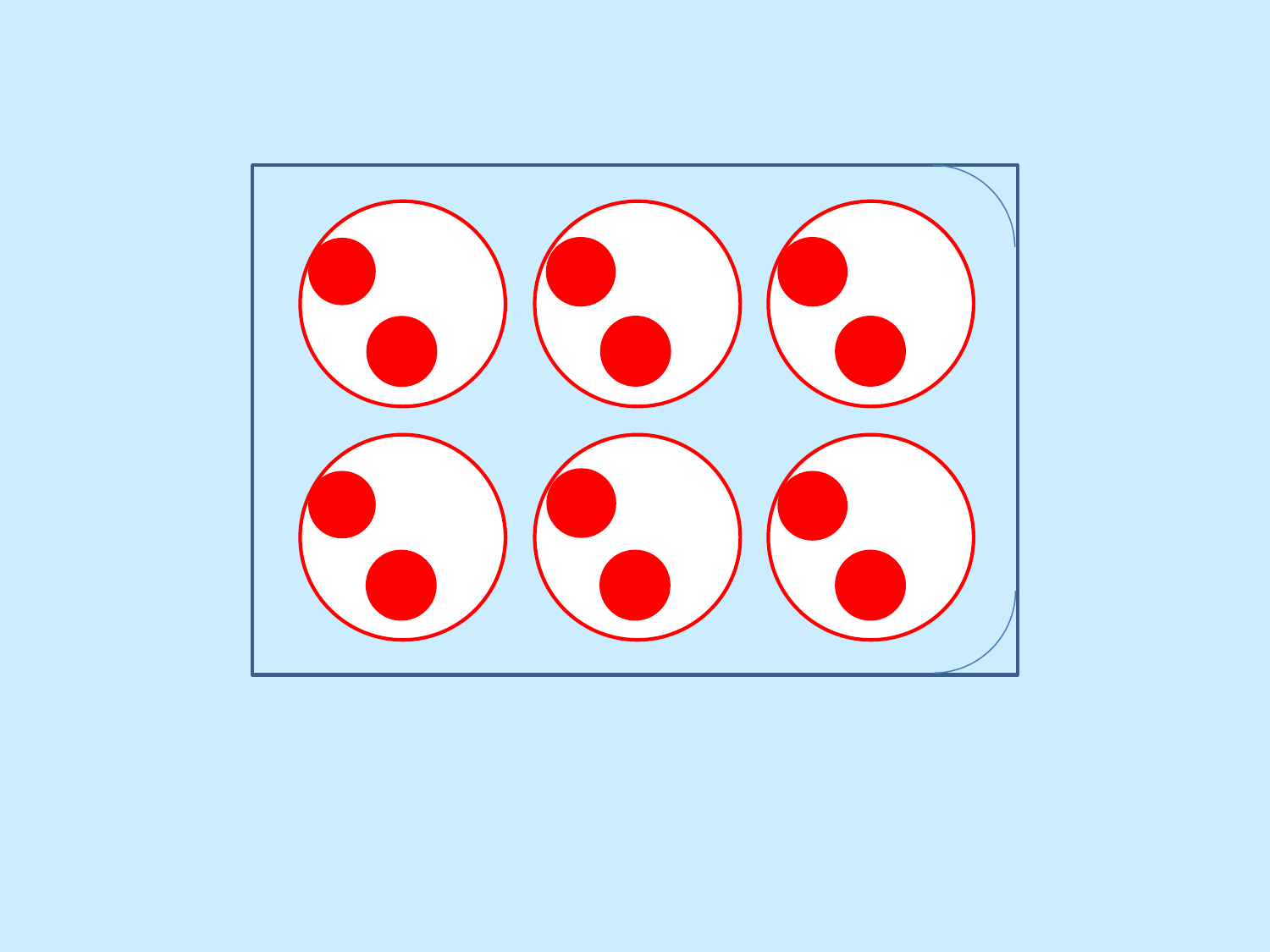

## Slide 3
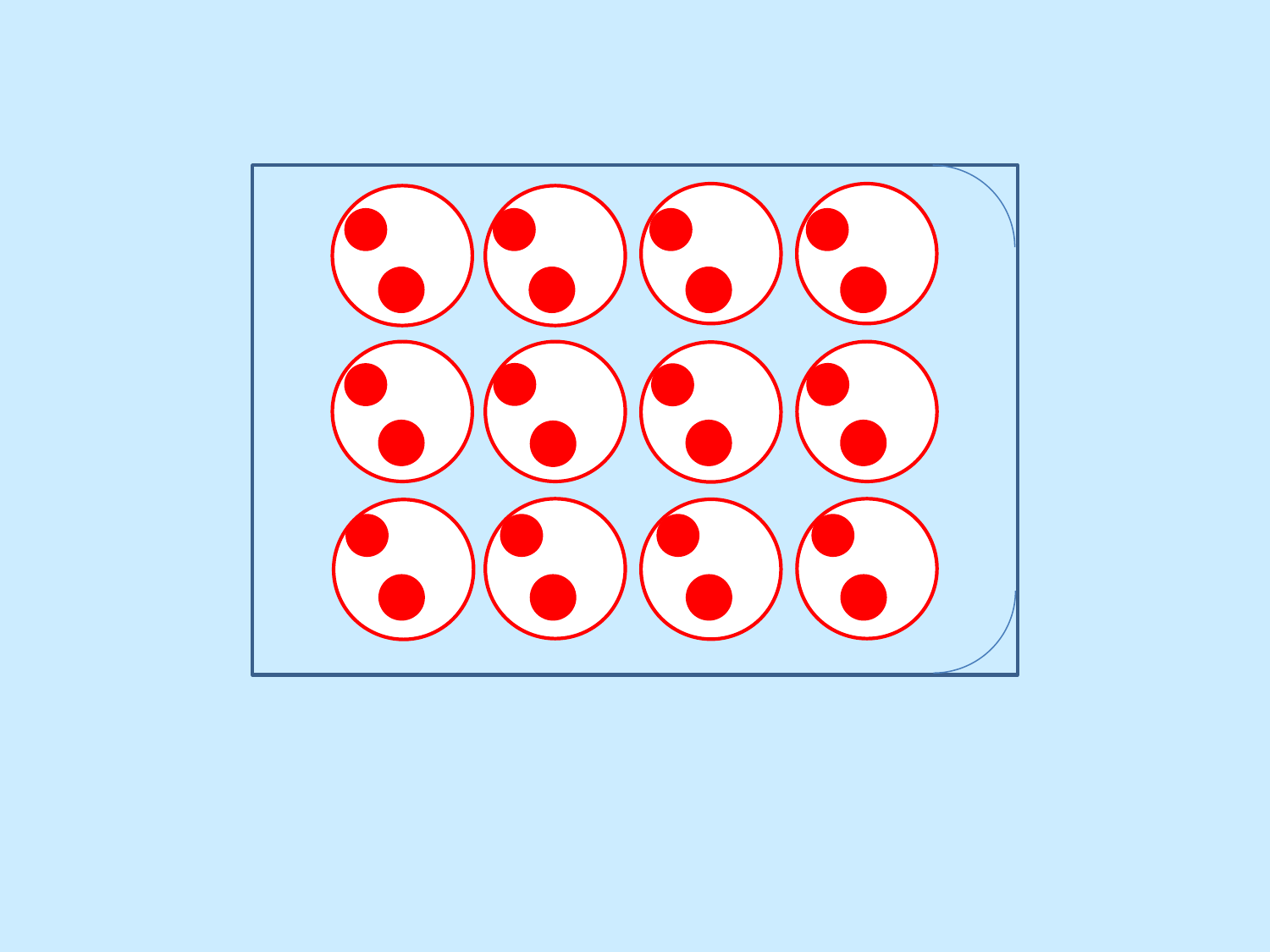

Supplement: Supplementary file 1 [file ijerph-19-10920-s001.zip › ijerph-1873904-supplementary/Supplementary Materials/visual stimuli.pptx]
